# The future is in the numbers: the power of predictive analysis in the biomedical educational environment

**DOI:** 10.3402/meo.v21.32516

**Published:** 2016-07-01

**Authors:** Charles A. Gullo

**Affiliations:** Office of Medical Education, Joan C. Edwards School of Medicine, Marshall University, Huntington, WV, USA

**Keywords:** big data, MLR analysis, prediction analysis, national standardized examinations

## Abstract

Biomedical programs have a potential treasure trove of data they can mine to assist admissions committees in identification of students who are likely to do well and help educational committees in the identification of students who are likely to do poorly on standardized national exams and who may need remediation. In this article, we provide a step-by-step approach that schools can utilize to generate data that are useful when predicting the future performance of current students in any given program. We discuss the use of linear regression analysis as the means of generating that data and highlight some of the limitations. Finally, we lament on how the combination of these institution-specific data sets are not being fully utilized at the national level where these data could greatly assist programs at large.

The era of big data is here to stay. The advent of social media, cloud computing, human genome mapping, and personalized medicine has created an industry that specializes in analyzing, capturing, sharing, and visualizing massive data sets. However, in biomedical science education, we seem to be stuck in the era of little data. We, as a community, do not regularly collaborate to create and share large data sets that directly benefit our students with anonymized internal data from our schools. Each and every biomedical program has a rich trove of data from student assessment milestones and admissions information that can inform student outcomes milestones and improve internal processes – much of these are closely guarded. What is currently lacking is the combined approach that allows for rich interrogation of large data sets from schools with similar program outcomes across the country (e.g., allopathic medical, osteopathic medical, pharmacy, or others that require national licensing examinations).

The marketing and business world is well aware of the potential of mining big data for predictive purposes – the vast majority of it tied to the purpose of getting consumers to spend more money. Indeed, a number of companies base their business model on providing this service. Predictions are valuable for medical, pharmacy and nursing schools for forecasting student outcomes on future standardized national exams. By utilizing existing data, these predictions can be of great value to admissions committees when determining their next cohort of students. Considering the large amount of financial resources and time that students invest in gaining a medical degree, using internal data sets to predict student performance is significant and worthwhile. Furthermore, as residency programs become more competitive with limited spots; students are acutely aware of the need to perform well in national exams. In addition, data that could be potentially useful for guiding medical admissions committees or for assisting medical administrators in identification of students who are likely to perform poorly in the future often remain untapped, unmined and limited to within the confines of a single institution.

Here, I describe a process we have used at the Joan C. Edwards School of Medicine (JCESOM) to assist our medical education team in 1) analyzing data sets and creating variables for predictive analysis, 2) using data-driven approaches for predicting student outcomes and 3) developing a process for the continuous evaluation of student performance. This process involves capturing of student data that exist in several locations, curating it for analytical analysis, performing step-wise linear regression, and establishing an internal visualization tool for easy interrogation of the data (see Fig. 1 for schematic overview).

**Fig. 1:**
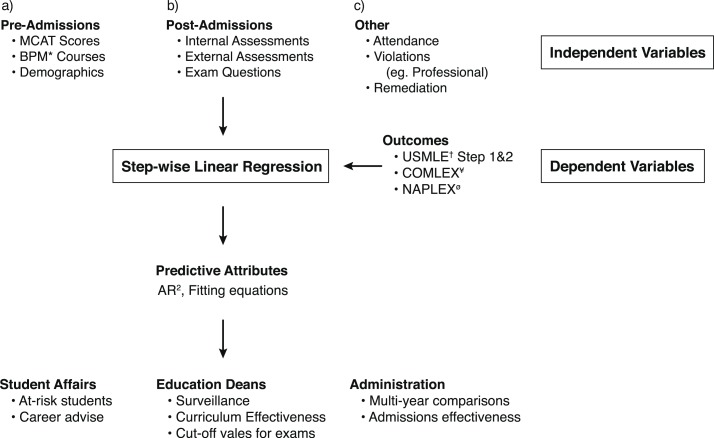
Schema of biomedical student prediction analysis. This figure represents the steps that were used at the JCESOM to predict students who would most likely struggle on the USMLE standardized exams. This includes the identification of dependent and independent variables, the linear regression data generated, and the end users of these data in a medical school environment. *Represents undergraduate Biology, Physics, and Math scores; ^†^represents United States Medical Licensure Exams; ^¥^represents the Comprehensive Osteopathic Medical Licensing Examination; and ^ø^represents the North American Pharmacist Licensure Examination.

## Discussion/results

Generalizations can be made of the predictive nature of the pre-matriculation information such as MCAT (Medical College Admission Test) and undergraduate GPA (Grade Point Average) scores in their ability to predict future outcomes such as performance on medical school licensure exams. It is generally assumed that high GPA in science and math and high MCAT scores correlate positively to strong performance on USLME (United States Medical Licensing Examination) Step 1(1–5). In addition, it is widely assumed that students who do well in internal exams will do well in national assessment exams, but this may not always be the case (6, 7). Either way, we advocate an approach where each program mines its own data and looks at as many variables as possible because it is likely that a positive correlation can be made with some variables but not with others. This is what we did at the JCESOM which, like many programs across the country, has a large number of potential variables to use (8).

It is my goal here to provide the overall process we recommend for establishing a data-driven approach to predicting future outcomes in national examinations. The first step involves defining what outcomes are most important to your program (examples include USMLE, Comprehensive Osteopathic Medical Licensing Examination (COMLEX), or North American Pharmacist Licensure Examination (NAPLEX)). Defining the outcome allows one to consider which independent variables are most likely to affect these outcomes the greatest. The outcome itself is the dependent variable in this type of analysis while the academic milestones that are used in the order in which they occur in the journey of the student, represent the independent variables. We chose two outcomes, both USMLE Step 1 and Step 2, based on the importance of these exams both as a graduation requirement and in the residency match.

Here at the JCESOM, we considered both pre-admission and in-class assessment data when reviewing the source of our independent variables. We identified a total of 22 pre-admissions data points for consideration, 2 data points from year one exams and 13 variables from year two examinations. Our prediction analysis involved the use of step-wise multivariate linear regression (MLR) in which variables were analyzed one at a time and only used if their coefficients were positive and the addition of the coefficient strengthened the predictive model. Variables that were not significant or additive in their predictive capacity were discarded. This method is useful for programs that have access to a wide variety of student data including those collected at the admissions stage, during the various assessment phases of the curriculum, and even data collected after students graduate and move onto residencies or the workplace. As we chose outcomes from the end of the second year (Step 1) and the end of the third year (Step 2CK), we specifically included data from milestones that occurred during the preclinical years. Another rich source of data from student affairs offices such as professionalism transgressions, attendance and clinical performance indicators can be useful if these data contain an objective measure.

It is important to note before continuing, that although we have come up with certain variables that predict well in our environment, these data are institution-specific and may not be the same for different schools. However, if outcomes are similar and the examination/milestone are the same (or similar), variables are likely to yield generalizable predictions from program to program. We have found that pre-admissions variables are less predictive than in-house curricular variables such as performance on the student's first exam in medical school when predicting performance on Step 1 exam. Although these findings may be replicated by other medical schools, the exact nature of the influence on performance is likely to differ.

The value of performing MLR analysis is that it allows one to determine the influence predictors have over a specific outcome. This value is referred to as the adjusted R^2^ (AR^2^). We found, for example, that pre-admissions data had a low predictive value of about 0.12 (or 12%) whereas all exams taken in the first year could predict about 38% of the variance in the Step 1 exam (8). As one starts adding more available data, the strength of the predictions improves and is especially so the closer the independent variables approach the dependent variables in time. We found that the AR^2^ value for the sum of all the MS2 exams was 53% which was significantly higher than that of the sum of the MS1 exams. This overall increase will most likely be replicated in data from other programs as well, but the exact values are likely to be significantly different. Our strongest predictive capabilities for Step 1 performance was achieved with a combination of the practice Step 1 exam and two basic science miniboards which gave an overall AR^2^ of 77%. In terms of Step 2 prediction, our strongest predictive capabilities resulted when we used the Step 1 results in addition to four clinical mini board results (AR^2^ of 62%).

The utility of the actual AR^2^ values are limited, but the generated equations containing the coefficients are very useful for fitting new data into existing linear regression functions. For example, the data we used for the aforementioned predictive analysis spanned 5 years’ worth of data. The AR^2^ values from these training data sets were helpful in determining the power that a set of variables had in predicting an outcome over background. What is more important, however, is the formula for those predictions that can be applied to current student data. An equation is determined for each prediction that is made up of coefficients that can be used to fit new data. For example, for the Step 2 predictions, the variable that contributed to the most robust predictions, a regression model, is generated with the following equation: Y=BO+0.31(X1)+0.9(X2)+0.11(X3)+0.07(X4)+0.15(X5); where BO represents the Y intercept and X1–X5 refers to the five independent variables used (8). The coefficients are created for each variable and this prediction equation can now be used to estimate the independent variable (Step 2) with data outside of the period used to fit the data (e.g. current students with new exam data). What this means, is that data from programs that are outside of the original source of the data can be potentially used to create the regression and may be fit into the model as long as their dependent variables are the same and the independent variables are similar (or adjustments can be made if they do differ).

To summarize, data from a defined period of time (we used 5 years’ worth of student exam and pre-admissions data) should be used to create an initial regression model. A step-wise approach allows one to walk through all relevant independent variables one at a time until a set of variables that allows for robust prediction can be determined in what can be thought of as a calibration set. AR^2^ values can be determined for each step and those that are statistically valid can be kept. An equation (the best fit of a line) is created where each variable is assigned a coefficient that allows new data to be used to reconstruct the prediction. There are a number of assumptions that need to be tested before one uses the data at hand including the need to show that the independent variables are truly independent of one another or multicolinear. However, most robust statistical packages such as SPSS^®^ (IBM^®^ SPSS^®^, Armonk, NY) and MatLab^®^ (The Mathworks^®^, Natick, MA) are designed to assist the user in testing these assumptions.

Outcome measures are important for any program, and prediction of performance on USMLE, COMLEX, or NAPLEX is valuable for a number of reasons. First, students spend significant time preparing for these exams, and providing students with their predicted scores is likely to be useful for this preparation. To this end, individualized data can be garnered for each student using the MLR formulas as discussed above. In fact, we use our regression models to compute students’ predicted scores for Step 1 and Step 2 at various stages along their preclinical journey. Although, we do not distribute the data to all students at every time point, we do use this and other data when counseling at-risk students. Besides students, administrators may have an interest in using these data to evaluate the overall quality of the education process. For example, it can be very useful to determine the numbers of students who are at risk using the MLR data at the end of the first year and second year and comparing these numbers over time. This can be very helpful when new curricular changes occur. These data are also very useful for the review of admissions criteria or to challenge/confirm assumptions about an entering class and their likelihood to do well in their future national exams.

## Conclusions

We have discussed the use of MLR for data-driven discovery work within a single organization. It would be beneficial for biomedical schools with similar outcomes to pool anonymized data together and test robust predictive models for admissions and curriculum review committees. It is also very likely that the output from the much larger data sets would be easier to validate due to the large sample sizes and thus be more robust. One could easily imagine a national database with de-identified data from all schools across the country (e.g., for medical, dental, or pharmacy, programs) where any program office from a participating school could mine the data for its own student attributes before deciding on what cutoffs they should use for admitting matriculants. One might envision a dataset of USMLE Step 1 and 2 scores and post matriculation variables for the past 10 years that medical school administrators could use to assist them in their future remediation plans for at-risk students. One could indeed argue that there are too many confounding variables that would restrict the use of this data, but much of this can be overcome with the use of non-parametric data analysis and other statistical tools that minimize the differences in the independent variables. At the very least, demographic data such as gender, age, biomedical background and many others would be readily comparable and usable in such data sets with minimal interference. Regardless, the benefits of these large discipline-specific data sets will certainly create more opportunities for existing programs.
